# The usefulness of contrast echocardiography in the evaluation of cardiac masses: a multicenter study

**DOI:** 10.1186/s12872-024-03708-2

**Published:** 2024-01-13

**Authors:** Qingtao Wang, Bing Wang, Xiaofeng Zhang, Xin Zhong, Shuai Chang, Jinbo Yang, Jian Liang, Qiangqiang You, Heng Zhou, Jiaqi Zhang

**Affiliations:** 1 Department of Cardio-Thoracic Surgery, Xiangyang No. 1 People’s Hospital, Hubei University of Medicine, No 15, Jiefang Avenue, Xiangyang, 441000 China; 2 Department of Ultrasound, Xiangyang No. 1 People’s Hospital, Hubei University of Medicine, No 15, Jiefang Avenue, Xiangyang, 441000 China; 3 Department of Ultrasound, First Affiliated Hospital of Guangxi Medical University, Nanning, 530021 China; 4 Department of Ultrasound, Hunan Provincial People’s Hospital, The First Affiliated Hospital of Hunan Normal University, Changsha, 410002 China; 5 Department of Ultrasound, First Affiliated Hospital of University of South China, Hengyang, 421001 China

**Keywords:** Cardiac mass, Contrast echocardiography, Thrombi, Benign lesion, Malignant lesion

## Abstract

**Background:**

Cardiac masses can encompass a variety of conditions, such as tumors, thrombi, vegetations, calcific lesions, and other rare diseases. Treatment and management of these types of cardiac masses differ considerably. Thus, accurately distinguishing among thrombi, benign tumors, and malignant tumors in the heart is of great importance. Contrast echocardiography (CE) has emerged as a promising technology. Although published guidelines suggest that CE can enhance image quality and assist in differentiating between benign and malignant lesions, most studies on CE diagnosis of cardiac masses are limited to case reports or retrospective/small-sample-sized prospective cohorts. This study aims to evaluate the diagnostic accuracy of CE in patients with suspected cardiac masses and address the insufficient evidence for differential diagnosis using CE.

**Methods:**

Between April 2018 and July 2022, a prospective multicenter study was conducted, which included 145 consecutive patients suspected to have cardiac masses based on transthoracic echocardiography. All patients underwent CE examinations. The echocardiographic diagnosis relied on qualitative factors such as echogenicity, boundary, morphology of the base, mass perfusion, pericardial effusion, and motility as well as quantitative factors such as the area of the masses and the peak intensity ratio of the masses to adjacent myocardium (A1/A2).

**Results:**

The final confirmed diagnoses were as follows: 2 patients had no cardiac mass, 4 patients had pseudomass, 43 patients had thrombus, 66 patients had benign tumors, and 30 patients had malignant tumors. The receiver operating characteristic (ROC) analysis indicated that an optimal A1/A2 cutoff value of 0.499 distinguished a cardiac tumor from a thrombus, with AUC, sensitivity, specificity, PPV, and NPV of 0.977, 97.9%, 90.7%, 95.9%, and 95.1%, respectively. The optimal A1/A2 cutoff value of 1.583 distinguished a cardiac tumor from a thrombus, with AUC, sensitivity, specificity, PPV, and NPV of 0.950, 93.3%, 93.9%, 87.5%, and 96.9%, respectively.

**Conclusions:**

Combined with qualitative and quantitative analyses, CE has the potential to accurately differentiate among different types of cardiac masses.

## Introduction

Since the advent of echocardiography, researchers have focused on cardiac masses. These masses can be divided into non-neoplastic masses (such as thrombi, vegetations, calcifications, or other rare conditions), pseudotumors (lesions not originating from a neoplastic transformation of a specific cell type), benign tumors, or malignant tumors. Non-neoplastic masses account for 75% of all cases [1, 2]. Although cardiac tumors are rare, primary cardiac tumors have a prevalence of 0.001–0.03%, while metastatic cardiac tumors occur 10–1,000 times more frequently (2.3–18.3%) [3–5]. Primary cardiac tumors are classified based on histological characteristics into benign or malignant. A previous study indicated that the distribution of cardiac tumors was 34% in the left atrium, 26% in the right atrium, 6% in the left ventricle, 7% in the right ventricle, and 27% in other locations [6].

Cardiac masses located in any chamber adjacent to large blood vessels or pericardium may require treatments, such as surgical removal or chemoradiotherapy, depending on the histopathological type, extent of invasion, and patient risk stratification [7]. Early detection and accurate differentiation of cardiac masses can lead to prolonged survival and improved quality of life for affected patients. Several imaging modalities are used to assess cardiac masses; these modalities include transthoracic echocardiography (TTE), transesophageal echocardiography (TEE), cardiac magnetic resonance (CMR), positron emission tomography (PET), computed tomography (CT) [8], CT-PET [9], etc. [10–12]. However, no guidelines or consensus have been established on the best diagnostic approach due to the diversity of cardiac masses. A recent comprehensive review suggests that TTE is typically the first choice for cardiac mass examination, and CMR provides high-resolution imaging for further evaluation if a mass is suspected. PET can be useful for staging malignancies and guiding biopsy location [13].

TTE is a valuable tool for determining the presence, size, shape, echogenicity, mobility, attachment point, and hemodynamic effects of cardiac masses and has a sensitivity of 93%. However, TTE may not be sufficient in some cases where image quality is suboptimal or the echoes are complex. Accurate differentiation between benign and malignant tumors using TTE can be challenging, with an accuracy of less than 70% [3, 14, 15]. To address these limitations, contrast echocardiography (CE) has emerged as a promising technology in recent years. Although published guidelines suggest that CE can improve image quality and aid in differentiating between benign and malignant lesions, most studies on CE diagnosis of cardiac masses are case reports or retrospective/small-sample-sized prospective cohorts [15–17]. The present study aimed to evaluate the diagnostic accuracy of CE in patients with suspected cardiac masses and address the insufficient evidence for differential diagnosis using CE.

## Materials and methods

This prospective study was conducted in four tertiary hospitals in China including First Affiliated Hospital of Guangxi Medical University, Hunan Provincial People’s Hospital, First Affiliated Hospital of University of South China and Xiangyang No. 1 People’s Hospital, Hubei University of Medicine.

### Study participants

Adult patients who underwent TTE between April 2018 and July 2022 and were suspected to have cardiac masses were included in this consecutive cohort study. Exclusion criteria were allergies to albumin, blood products, or ultrasound enhancing agents. Patients with severe heart failure (New York Heart Association Class IV), severe arrhythmia, respiratory failure, severe liver or kidney dysfunction, or mental illness or epilepsy were also excluded.

### Echocardiographic image acquisition

Each patient underwent echocardiographic examinations in the left lateral position by using a Philips iE33 ultrasound system (Philips Medical Systems, Bothell, WA, USA) and a TTE probe (S5–1, 1–5 MHz) by an echocardiographist with over 10 years of TTE experience at each center. All images and measurements were obtained in accordance with the echocardiography guideline [18]. Following the TTE examination, all patients underwent CE according to the most recent published guidelines [19].

### CE protocol

The study protocol was designed in accordance with the most recent guideline for CE [20]. Commercially available ultrasound enhancing agents (SonoVue; Bracco, Plan-Les-Ouates, Switzerland) were utilized during CE. The left ventricular opacification (LVO) mode was activated with a low mechanical index of 0.2 and 30-Hz frame rates. Subsequently, 0.8 mL of prepared ultrasound enhancing agents were rapidly injected via peripheral vein, followed by a slow (10–20 s) 3–5 mL saline flush as necessary to achieve optimal delineation of the left ventricular cavity and cardiac masses. Morphological and hemodynamic features of cardiac lesions were observed and digitally saved in this mode. The myocardial CE (MCE) mode was activated with a very low mechanical index of 0.08 and 30-Hz frame rates. After the left ventricle and myocardium were filled, the ultrasound enhancing agents were continuously infused with a dedicated Vueject R syringe pump (Bracco, Milano, Italy) at a rate of 1 mL/min. Intermittent flash technique (high mechanical index of 1.0) was employed to destroy the microbubbles. High mechanical index ultrasound impulse was transmitted between 5 and 10 frames to destroy the microbubbles. Perfusion was verified after contrast replenishment following the impulse to prevent false-positive readings caused by saturation artifact. The imaging results of the masses and adjacent normal myocardium before and after the flash were saved.

### Echocardiographic image analysis

**Fig. 1:**
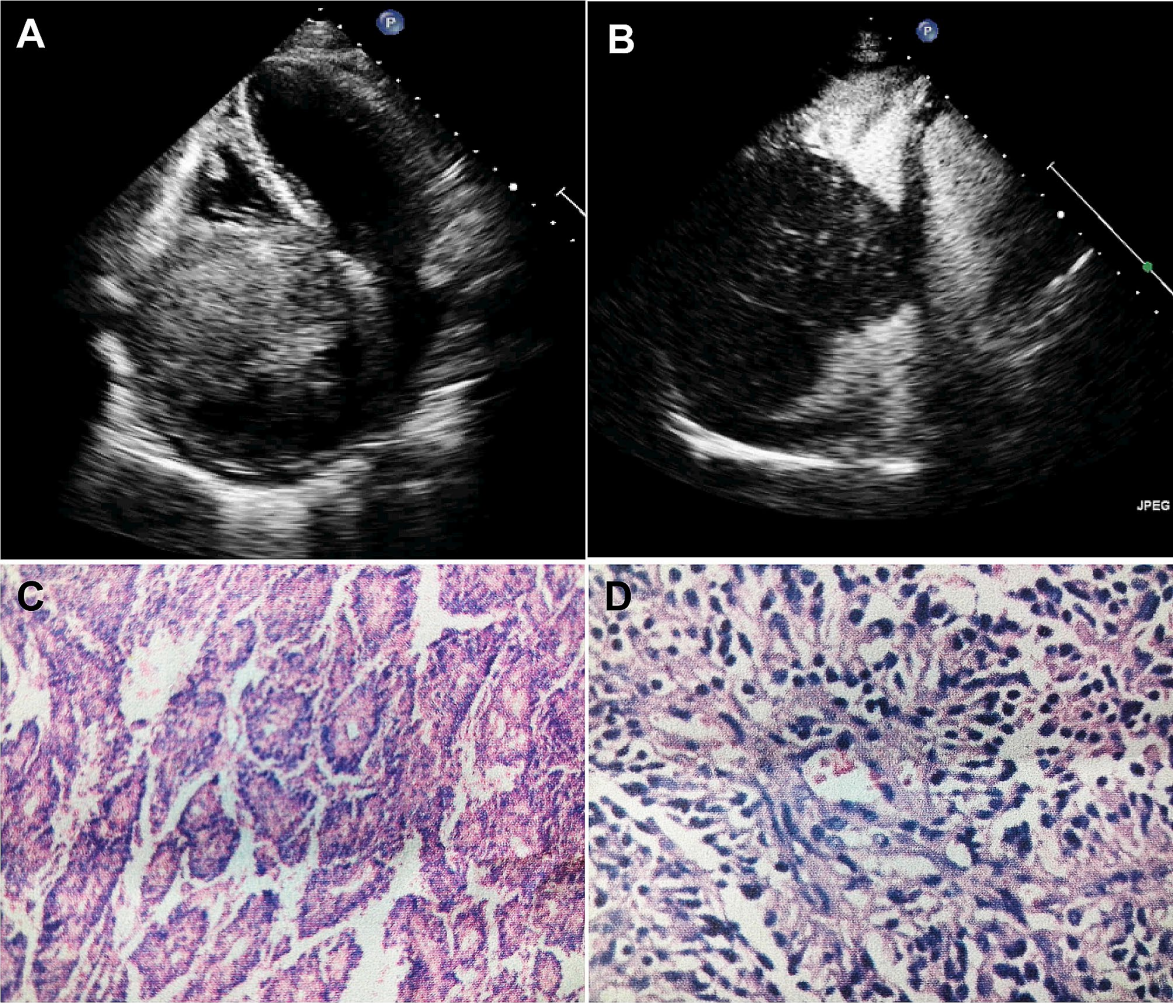
(**A**) A slightly strong echogenic mass can be seen in the right atrium, and it almost fills the right atrium. The internal echo is uneven and appears to adhere to the anterior wall of the right atrium with a wide base, with little amplitude swing with the cardiac cycle. (**B**) Contrast-enhanced signal can be seen in the slightly stronger echogenic mass above the right atrium. (**C, D**) Immunohistochemistry: VIM (+), CD31 (+), FVM (+), FLI1 (+), D2-40 (-), Ki-67Li about 40%, final diagnosis through histological examination as angiosarcoma (right atrial)

**Fig. 2:**
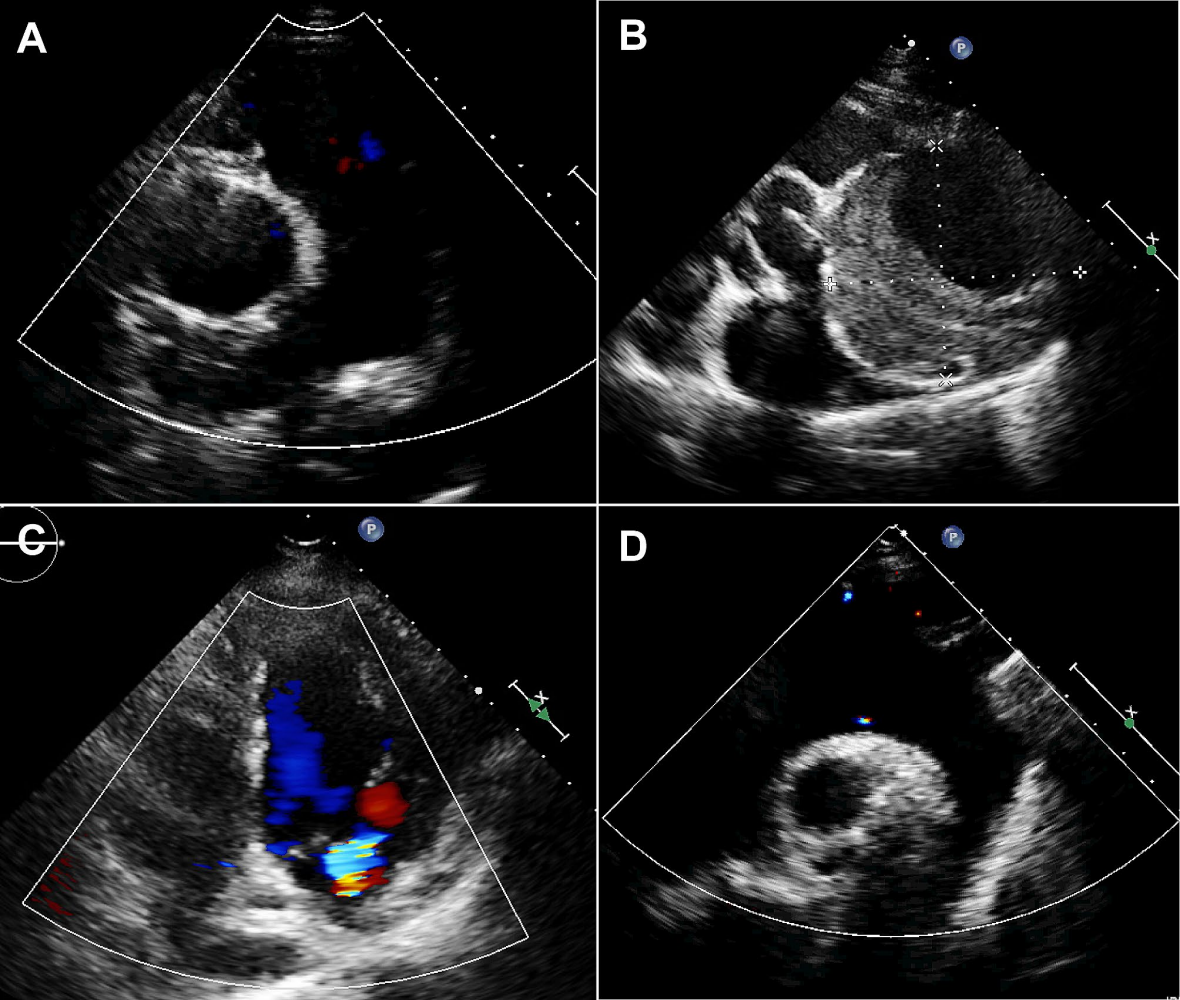
(**A, B**) A solid-cystic mass can be seen outside the left coronary sinus of the aorta in echocardiography. (**C**) No blood flow in the isoechoic area of the mass. (**D**) The contrast agent enters the cystic cavity after continuous interruption, and the enhancement in the isoechoic area is not obvious; it is ultimately diagnosed as a huge coronary artery aneurysm and concurrent thrombosis

**Fig. 3:**
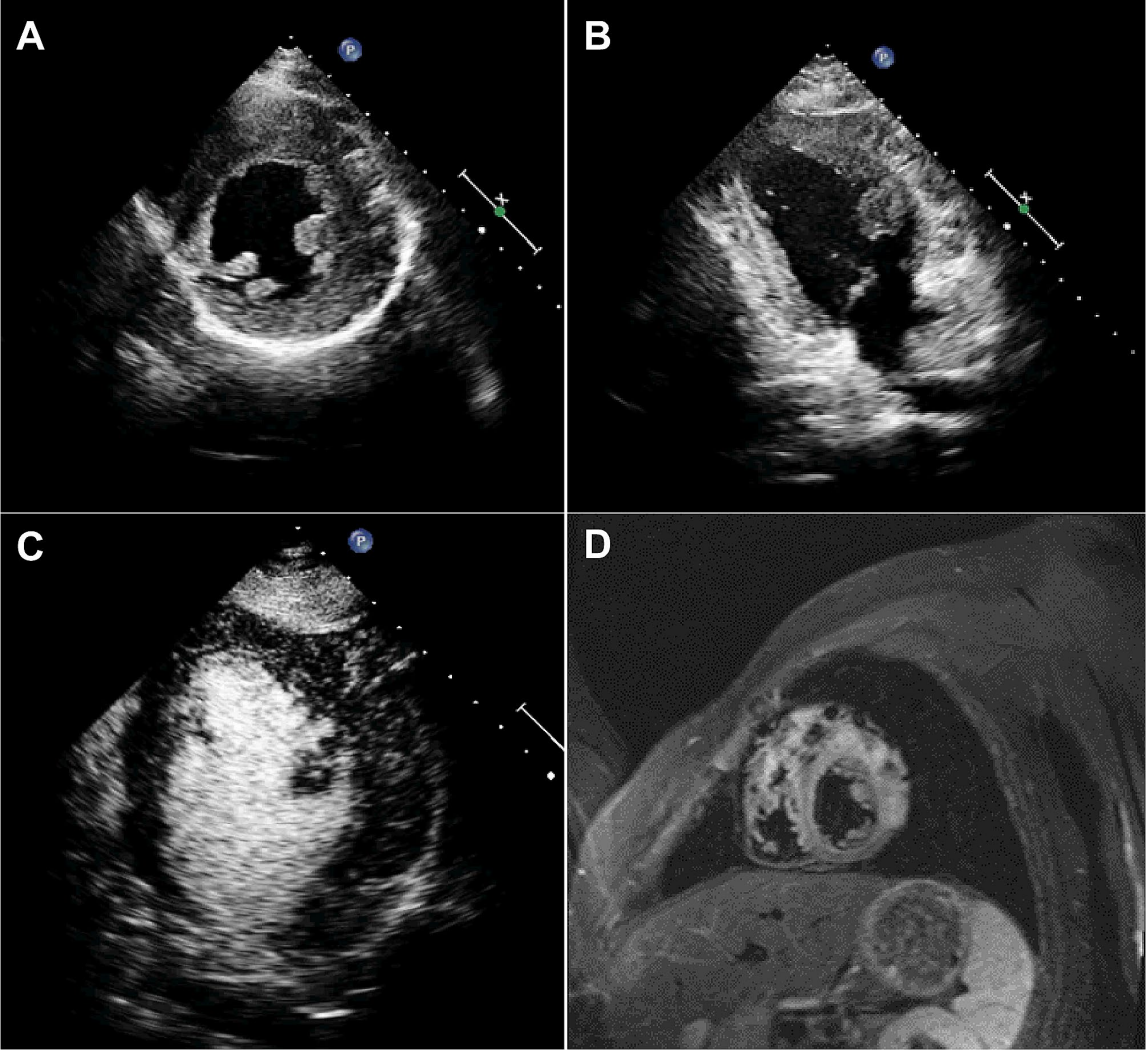
(**A, B**) Abnormal masses can be seen in the epicardial layer of the lateral wall of the left ventricle, with strong echoes and dark areas separated by radial ribbons. The boundary with normal myocardial echo is still clear, and the left ventricular normal myocardial hypertrophy is mild. (**C**) The left ventricular wall is significantly thickened, and the abnormal echogenicity of the left ventricular lateral wall appears to be accompanied by contrast agent echo. (**D**) MRI revealed uneven thickening of the myocardium, local nodular formation, and diffuse edema, ultimately confirmed by surgery as myocardial cavernous hemangioma

The study used qualitative and quantitative analyses. For patients with an echocardiographic suspicion of cardiac masses, qualitative analysis included observing echogenicity (uniform/non-uniform), boundary (well-demarcated/not well-demarcated), base morphology (narrow with peduncle/narrow with notch/broad), mass perfusion (no perfusion/mild perfusion/intense perfusion) [21], motility (absent/present), and pericardial effusion (absent/present) [22, 23]. Two physicians with 10 years of experience in echocardiography jointly made a diagnosis based on the above qualitative indicators. Quantitative analysis was conducted using QLAB software (version 13.0; Philips Medical Systems, Andover, MA, USA). The area of the masses was measured when the long maximum diameter was visible, and the peak intensity of the masses and adjacent myocardium were measured as A1 and A2, respectively, with a ratio of A1 to A2 to differentiate between malignant and benign tumors (Figs. 1, 2 and 3).

### Follow-up and validation

All patients were prospectively followed up until March 1, 2022 to determine all-cause mortality by reviewing their medical records, conducting telephone interviews, and performing outpatient examinations every 6 months. Three types of cardiac masses were identified. (I) Pseudomass is defined as a variant or prominent normal structure, including Eustachian valve or Chiari network, Crista terminalis, and Coumadin ridge. The diagnosis was confirmed using CMR, and no morphological changes were observed in follow-up imaging [24]. (II) Thrombus is defined as a distinct mass of echoes visible throughout systole and diastole. The diagnosis was confirmed based on one of the following two criteria: (i) a significant reduction in size or complete resolution after anticoagulation therapy, with confirmation of thrombus upon follow-up TEE or CT; or (ii) pathological confirmation [25]. (III) All tumors were confirmed by surgery or biopsy and classified as benign or malignant based on the 2015 World Health Organization classification of tumors of the heart and pericardium [26].

### Statistical analysis

Continuous parameters were presented as mean ± standard deviation, while non-normally distributed parameters were shown as median (interquartile range, IQR). Independent sample t-test was used to evaluate differences in continuous parameters among groups, while Mann–Whitney U test was used for non-normally distributed parameters. Pearson’s Chi-squared test or Fisher’s exact test was used to compare categorical parameters among groups. Receiver operating characteristic (ROC) analysis was used to determine the differentiating capacity of variables for cardiac masses. Youden’s J statistic was used to identify the optimal cut-off value. The area under the receiver operating characteristic curve (AUC), accuracy, sensitivity, specificity, positive predictive value (PPV), and negative predictive value (NPV) were calculated. The level of statistical significance was set at *P* < 0.05.

## Results

### Population characteristics

**Fig. 4:**
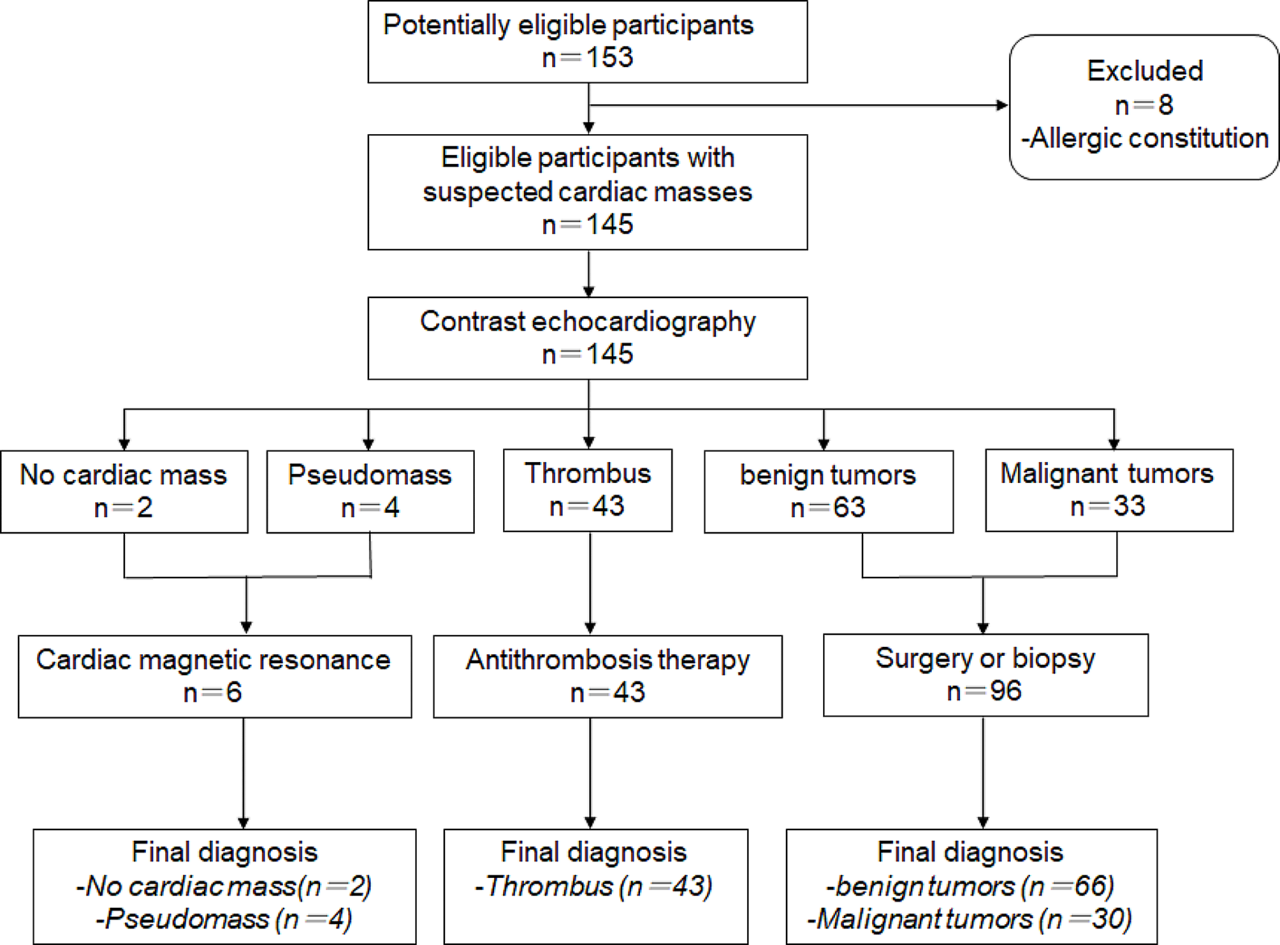
Diagnostic process based on qualitative characteristics such as echogenicity, boundary, base, mass fusion, motility, and pericardial fusion

Between April 2018 and July 2022, a total of 49,354 TTEs were performed at six departments, of which 153 (0.31%) examinations were conducted on patients with suspected cardiac masses. Eight patients with allergic constitution refused CE (Fig. 4). A total of 145 patients with a median age of 59.4 years (IQR: 51.2–63.9 years) and including 90 (62.0%) men were enrolled. Table 1 summarizes the baseline demographic and clinical characteristics of all patients. Of the 145 patients, two did not have any cardiac masses, four had a cardiac pseudomass, 43 had a cardiac thrombus, 66 had a benign tumor, and 30 had a malignant tumor. These findings indicated that the history of previous cardiovascular disease and malignancy varied significantly among the four groups.

**Table 1:** Characteristics of the population

	No cardiac mass (n = 2) and Pseudomass (n = 4)	Thrombus (n = 43)	Benign tumor (n = 66)	Malignant tumor (n = 30)	*P* value
Age, mean (SD), years	59.2 (13.6)	54.6 (15.4)	53.8 (11.5)	64.3 (10.3)	0.163
Sex (Male/Female)					0.322
Male	4	23	46	17	
Female	2	20	20	13	
Symptom					0.993
Asymptomatic	1	7	12	5	
Dyspnea	2	15	22	8	
Chest pain	2	9	13	7	
Palpitations	0	6	12	7	
Others	1	6	7	3	
History of cardiovascular disease	5	38	31	17	< 0.001
History of malignant disease	0	3	6	27	< 0.001
Localization					0.154
Left ventricle	3	10	8	5	
Left atrium	1	15	26	4	
Right ventricle	1	6	10	9	
Right atrium	0	7	15	7	
Others	1	5	7	5	

SD, standard deviation

Three cases of cardiac pseudomass were attributed to the hypertrophy of the interatrial septum, while one case was due to the hypertrophy of the papillary muscle. Anticoagulation therapy was administered to all patients diagnosed with a cardiac thrombus, and none underwent pathological analysis. Solitary thrombi were observed in all cases. Among the 43 patients with thrombi, 72.1% (31/43) experienced dissolution, and 27.9% (12/43) had a significant reduction in thrombus volume. Benign tumors were confirmed through surgery (60/66) and biopsy (6/66). Malignant tumors were confirmed through surgery (9/36) and biopsy (27/36).

Following the administration of contrast enhancement (CE), two investigators diagnosed cardiac masses based on qualitative characteristics such as echogenicity, boundary, base, mass fusion, motility, and pericardial fusion. The results revealed that out of 145 cardiac masses, 140 were consistent with the final diagnosis, yielding a diagnostic accuracy of 96.6%. Of the 140 consistent diagnoses, 2 cases had no mass, 4 were pseudomasses, 43 were thrombi, 63 were benign tumors, and 28 were malignant tumors. However, instances of misdiagnosis were recorded. Three benign tumors erroneously identified as malignant tumors, and two malignant tumors were misclassified as benign tumors (Fig. 4).

### Comparison and differentiation of cardiac tumors from thrombi

**Fig. 5:**
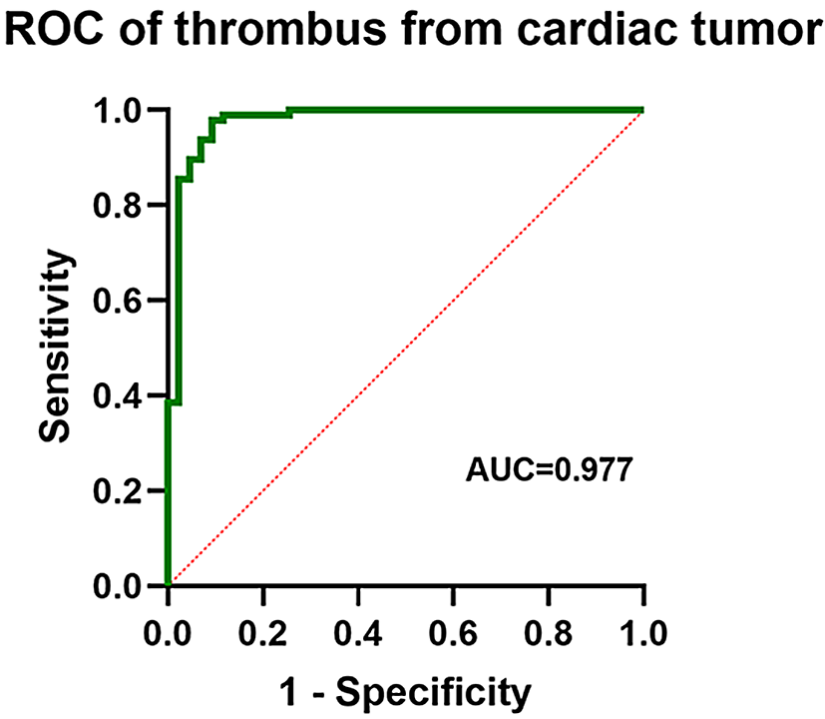
ROC curve for identifying thrombi and cardiac tumors by using quantitative analysis values A1/A2 of cardiac CE

The tumor group exhibited a significantly larger area, higher rate of non-uniform echogenicity, wider base, higher perfusion intensity, and higher A1/A2 compared with the thrombus group (*P* < 0.05, Table 2). When the cut-off value for A1/A2 was set to 0.499, the AUC for A1/A2 was 0.977 (95% CI: 0.947–1.000). The sensitivity, specificity, accuracy, PPV, and NPV were 0.979, 0.884, 0.957, 0.959, and 0.957, respectively (Fig. 5; Table 3).

**Table 2:** Comparison of echocardiographic parameters between thrombus and tumor

	Thrombus (*n* = 43)	Tumor (*n* = 96)	P value
Area, mean (SD), mm^2^	917.6 (386.7)	1,513.2(794.2)	< 0.001
Echogenicity			0.002
Uniform	29	37	
Non-uniform	14	59	
Boundary			0.254
Well-demarcated	31	59	
Not well-demarcated	12	37	
Base			< 0.001
Narrow with peduncle	0	32	
Narrow with notch	39	23	
Broad	4	41	
Mass perfusion			< 0.001
No perfusion	27	0	
Mild perfusion	11	54	
Intense Perfusion	5	42	
Motility			0.172
Absent	33	62	
Present	10	34	
Pericardial effusion			0.438
Absent	31	61	
Present	12	35	
Enhancement A1/A2,	0.39	1.20	< 0.001
median (IQR)	(0.20–0.76)	(0.83–1.57)	

CI, confidence interval; IQR, interquartile range; OR, odds ratio; SD, standard deviation. * variables entered into the multivariate regression included area, echogenicity, base, massperfusion, and enhancement A1/A2

**Table 3:** Comparison of diagnostic performance in differentiating thrombus from cardiac tumor and Malignant tumor from benign tumor

		Sensitivity	Specificity	AUC	Accuracy	PPV	NPV
Thrombus/cardiac tumor	A1/A2 (Cutoff value = 0.499)	0.979 (90.92-99.8%)	0.884 (74.3-96.1%)	0.977 (0.947-1.000)	0.9568 (0.936–0.994)	0.959 (0.943–0.981)	0.951 (0.947-1.000)
Malignant tumor/benign tumor	A1/A2 (Cutoff value = 1.583)	0.933 (77.9-99.2%)	0.939 (85.2-98.3%)	0.950(0.894-1.000)	0.9375 (0.921–0.984)	0.875 (0.841–0.978)	0.969 (0.962-1.000)

AUC, the area under the receiver operating characteristic curve; NPV, negative predictive value; PPV, positive predictive value

### Comparison and differentiation of malignant tumors from benign tumors

**Fig. 6:**
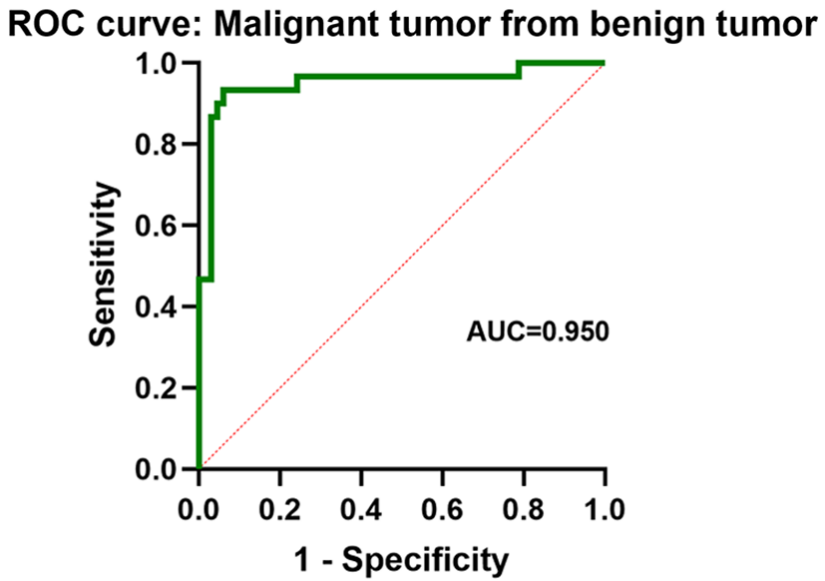
ROC curve for differentiating benign and malignant cardiac tumors using quantitative analysis values A1/A2 of cardiac CE.

Compared with the benign group, the tumor group exhibited a larger area, a higher rate of non-uniform echogenicity, an indistinct boundary, a wider base, presence of motility, and higher A1/A2 (*P* < 0.05, Table 4). The AUC for A1/A2 was 0.950 (95% CI: 0.894–1.000) when the cutoff value was set to 1.58. The sensitivity, specificity, accuracy, PPV, and NPV were 0.933, 0.939, 0.938, 0.875, and 0.969, respectively (Fig. 6; Table 3).

**Table 4:** Comparison of echocardiographic parameters between malignant tumor and benign tumor

	Benign tumor (*n* = 66)	Malignant tumor (*n* = 30)	P value
Area, mean (SD), mm	1,253.26 (659.25)	1,812.43 (713.59)	< 0.001
Echogenicity			
Uniform	34	3	< 0.001
Non-uniform	32	27	
Boundary			< 0.001
Well-demarcated	49	10	
Not well-demarcated	17	20	
Base			< 0.001
Narrow with peduncle	28	4	
Narrow with notch	20	3	
Broad	18	23	
Mass perfusion			0.045
Mild perfusion	42	12	
Intense Perfusion	24	18	
Motility			0.011
Absent	37	25	
Present	29	5	
Pericardial effusion			0.253
Absent	39	22	
Present	27	8	
Enhancement A1/A2, median (IQR)	1.07 (0.64–1.17)	1.48 (0.77–2.08)	< 0.001

CI, confidence interval; IQR, interquartile range; OR, odds ratio; SD, standard deviation

## Discussion

This study reports that the diagnostic performance of CE is notable in patients with suspected cardiac masses. CE exhibited high sensitivity and specificity in distinguishing cardiac tumors from non-neoplastic cardiac masses. It outperformed conventional TTE and showed comparable accuracy with pathological analysis in discriminating between malignant and benign tumors.

Cardiac masses pose a significant threat to patients, and improving their diagnostic efficiency is an essential objective for radiologists and cardiologists. The management of the diagnostic approach is also important for clinicians [4, 14]. Currently, TTE, TEE, cardiac CT and CMR are commonly used in diagnostic procedures. In the approach to cardiac masses, some echocardiographic parameters could provide good diagnostic accuracy if integrated in weighted and not weighted scores [9, 27]. CMR is the subject of intense research and exhibits excellent accuracy in differentiating cardiac thrombi from tumors and distinguishing between benign and malignant neoplasms in various retrospective and prospective studies. Although prospective studies have highlighted useful imaging characteristics such as tumor size, invasiveness, irregular border, and late heterogeneous gadolinium enhancement, they have been limited to qualitative or semi-quantitative analysis. Therefore, a diagnostic imaging technique with quantitative parameters should be developed to ease the burden on CMR and reduce the workload of pathologists [10, 12, 23].

TTE is still the primary imaging modality used to evaluate cardiac masses [15]. However, conventional TTE has limitations in accurately assessing the characteristics of cardiac tumors, particularly in differentiating benign from malignant tumors; it also heavily relies on the experience of the radiologist and cardiologists [28]. To overcome this limitation, CE has become an essential part of echocardiography because it can improve the accuracy of left ventricular ejection fraction measurement and provide quantitative analysis of cardiac masses. Kirkpatrick et al. [29] demonstrated the diagnostic utility of A1 and A2 values by using CE in cardiac masses in 2004; subsequent studies provided evidence for the differential diagnostic value of A1/A2. For example, Xia et al. [30] found a significant difference in A1/A2 between malignant and benign tumors, while Mao et al. [31] revealed that A1/A2 > 1 had a high diagnostic accuracy in differentiating benign masses from malignant metastatic tumors in a cohort study.

### Differentiation between cardiac tumors and thrombi

In this study, it was discovered that CE demonstrated remarkable accuracy in diagnosing intracardiac thrombi. When diagnosing a thrombus, setting A1/A2 with a cut-off value of 0.499 exhibited a specificity of 97.9% and a sensitivity of 88.4%. The A1/A2 value for the majority of thrombi was close to zero. However, in three cases, the A1/A2 values were significantly higher (1.39, 1.62, and 1.47) possibly due to fresh thrombi. In previous research, the loose texture of fresh thrombi and the ability of ultrasound-enhancing agents to enter from the periphery during CE resulted in a higher A1/A2. By contrast, old thrombi have a dense texture, and microbubbles of the ultrasound-enhancing agents cannot penetrate; as such, the A1/A2 values are near zero. Differentiating between fresh and old thrombi is crucial because fresh thrombi are more easily removed and less attached to the left ventricular wall; this structure makes them more brittle due to their collagen-poor organization. As a result, careful evaluation of the risk of fresh thrombus shedding is necessary.

Uenishi et al. [32] demonstrated another perfusion phenomenon, where ultrasound-enhancing agents did not penetrate the interior of the thrombus (81.8%, 27/33) or remained only at the periphery (12.1%, 4/33). They also found that the agents typically perfused the periphery (44.7%, 21/47) or even the entire cardiac tumor (48.9%, 23/47). However, additional samples are required to confirm the perfusion patterns observed in the present study and the findings of Uenishi et al. in the future.

### Differentiation between cardiac malignant tumors from benign tumors

This study demonstrates that CE could effectively distinguish between most benign and malignant tumors through qualitative and quantitative diagnostic methods. CE can enhance image quality and assess blood supply within tumors. Malignant tumors typically have abundant blood supply, and benign tumors have a sparse blood supply [33]. In previous studies, an A1/A2 cut-off value of 1.0 was utilized to differentiate malignant from benign tumors [29, 34, 35]. However, some benign tumors may have an A1/A2 value that is close to or slightly higher than 1, such as 1.32 in hemangioma, 1.08 in rhabdomyoma, 0.84 in fibroma, and 0.92 in hemangioma, and 1.06–1.15 in myxoma. Some malignant tumors contain necrotic tissues, which can result in an A1/A2 value of less than 1, as seen in 3.6% of cases in the study of Mao et al. The present study suggests that a cut-off value of 1.58 is better than 1 for differentiating malignant from benign tumors by using A1/A2. For less experienced radiologists, using A1/A2 with a cut-off value of 1.58 would result in good diagnostic accuracy. The size of the tumor area is beneficial in distinguishing between malignant and benign tumors, consistent with prior research.

This study possesses several strengths, including a novel diagnostic approach for distinguishing cardiac masses, a prospective study design, and a relatively large sample size. The use of a simple, rapid, and highly reproducible quantitative parameter (A1/A2) can greatly assist in clinical diagnosis, particularly for radiologists without extensive experience in using TTE to diagnose cardiac masses [36].

### Other modalities

In addition to TTE, transesophageal (TEE) echocardiography, cardiac CT, CMR, and 18Ffluorodeoxyglucose (18 F FDG)-PET have a complementary and mutually reinforcing role in assessing cardiac masses. TEE can serve as a valuable tool in diagnosing cardiac masses. Previous studies demonstrated that ultrasound-enhancing agents can enhance the diagnostic accuracy of cardiac thrombi during TEE for patients with atrial fibrillation [6]. Xia et al. also reported that combining TEE with CE can detect suspected cardiac masses and had an accuracy of 97.8–100%, particularly in distinguishing between benign and malignant lesions [30].

With the availability of various tissue characterization imaging sequences, CMR has distinctive advantages in noninvasively diagnosing cardiac masses. In a recent study involving 213 pediatric cardiac masses, CMR demonstrated the following diagnostic accuracies for cardiac tumors: 94% for fibromas, 71% for rhabdomyomas, and 50% for myxomas [37].

Cardiac CT may serve as an alternative to CMR, particularly in cases where other imaging techniques are non-diagnostic or contraindicated [38]. Cardiac CT is particularly useful for evaluating calcified masses compared with other imaging modalities. Previous studies demonstrated that the diagnostic accuracy of cardiac CT in predicting the malignant nature of cardiac masses could be more than 90% [9]. However, the use of cardiac CT has some limitations, including radiation exposure, a low risk of contrast-induced nephropathy, and a restricted soft tissue and temporal resolution in comparison with magnetic resonance imaging. Studies have suggested that cardiac CT can distinguish between cardiac tumors and thrombi [39]; however, further research with a large sample size is required to confirm this finding.

18 F-FDG PET/CT is confirmed as an extremely powerful tool to provide substantial information regarding the nature of cardiac masses. A recent study reported that the accuracy of 18 F-FDG PET/CT in predicting the benign or malignant nature of cardiac masses exceeds 91%. In particular, the study emphasized the value of PET in cases with inconclusive diagnoses following cardiac CT, specifically among patients exhibiting three or four abnormal CT findings. In these instances, the presence of all PET parameters below the specified cut-off values indicates a benign mass, while the identification of at least one abnormal PET characteristic reliably indicates malignancy [9].

### Limitations

This study has several limitations that need to be addressed. First, the participating hospitals were tertiary, which may have introduced selection bias because secondary hospitals typically treat thrombi with a well-demarcated boundary and low echocardiographic suspicion. Second, more cases of pseudomass should be included in future analyses because their low representation in the current study resulted in limited conclusions. Third, the recruitment period was short, and long-term follow-up may be necessary to determine whether A1/A2 can predict the prognosis for patients with cardiac tumors. Finally, the analysis did not explore the diagnostic performance of CE by less experienced radiologists, which may be an underlying confounder in this study.

## Conclusions

CE can be a promising tool in accurately differentiating cardiac masses by combining qualitative and quantitative analyses. However, additional studies with larger sample sizes are needed to validate these findings due to the limited sample size and the potential for underlying confounders.

## Data Availability

The data used to support the findings of this study are available from the corresponding author upon request.

## Abbreviations

AUC: Area Under Curve
CE: Contrast echocardiography
CMR: Cardiac magnetic resonance
LVO: Left ventricular opacification
MCE: myocardial contrast echocardiography
PPV: Positive predictive value
NPV: Negative predictive value
ROC: Receiver operating characteristic curve
TEE: transesophageal echocardiography
TTE: Transthoracic echocardiography
